# Development and validation of a delayed presenting clubfoot score to predict the response to Ponseti casting for children aged 2–10

**DOI:** 10.1007/s11751-018-0324-z

**Published:** 2018-11-15

**Authors:** T. R. Nunn, M. Etsub, T. Tilahun, R. O. E. Gardner, V. Allgar, A. M. Wainwright, C. B. D. Lavy

**Affiliations:** 1 CURE Ethiopia Children’s Hospital, Addis Ababa, Ethiopia; 2 University of York, York, UK; 3 Nuffield Department of Orthopaedics, Rheumatology and Musculoskeletal Sciences, Oxford, UK

**Keywords:** Clubfoot, Childhood, Delayed presenting, Score

## Abstract

The aim of the study was to develop a simple and reliable clinical scoring system for delayed presenting clubfeet and assess how this score predicts the response to Ponseti casting. We measured all elements of the Diméglio and the Pirani scoring systems. To determine which aspects were useful in assessing children with delayed presenting clubfeet, 4 assessors examined 42 feet (28 patients) between the ages of 2–10 years. Selected variables demonstrating good agreement were combined to make a novel score and were assessed prospectively on a separate consecutive cohort of children with clubfeet aged 2–10, comprising 100 clubfeet (64 patients). Inter-observer and intra-observer agreement was found to be greatest using the following clinically measured angles of the deformities. These were plantaris, adductus, varus, equinus of the ankle and rotation around the talar head in the frontal plane (PAVER). Measured angles of 1–20, 21–45 and > 45 degrees scored 1, 2 and 3 points, respectively. The PAVER score was derived from both the sum of points derived from measured angles and a multiplier according to age. The sum of the points was multiplied with 1, 1.5 or 2 for ages 2–4, 5–7 and 8–10, respectively. This demonstrated a good association with the total number of casts to achieve a full correction (tau = 0.71). A score greater than 18 out of 30 indicated a cast-resistant clubfoot. The score could be used clinically for prognosis and treatment, and for research purposes to compare the severity of clubfoot deformities.

**Electronic supplementary material:**

The online version of this article (10.1007/s11751-018-0324-z) contains supplementary material, which is available to authorized users.

## Introduction

Untreated, delayed presenting clubfoot remains a common problem in many low- or middle-income countries such as Ethiopia [1]. The prevalence is estimated to be as high as 1:500 in some Sub-Saharan African countries [2]. Our CURE infant Ponseti treatment programme currently covers approximately half of the population who are born with clubfoot in Ethiopia. Owing to the remoteness and limited access to services, we manage a large volume of children with untreated clubfeet. Clubfoot deformity is associated with reduced opportunities in life due to social exclusion, indignity, pain on walking on hard surfaces and hunger [3]. Additional local cultural implications overlay these factors making it an important condition to treat successfully. The goal of clubfoot treatment is to correct the deformity to give a painless, plantigrade, shoeable foot whilst maintaining as much movement and muscle power as possible [4, 5].

Up until 2014, our standard approach to the untreated clubfoot involved the use of joint-invasive procedures and bony resections. This was in an attempt to correct the foot in a single procedure which was convenient for family and social reasons. Following the success of Ayana and Klungsøyr [6] in Ethiopia, who treated delayed presenting clubfoot up to the age of 10 with Ponseti techniques, we changed our protocol and started manipulation and casting children instead. In our experience, this has been successful in the immature foot for the majority. It has the advantage of avoiding extensive surgical release with prolonged orthotic use, surgical morbidity and high recurrence rates in the presence of significant foot scaring.

The Pirani and Diméglio scoring systems, developed for the infant with a clubfoot, have been used by many authors in assessing delayed presenting clubfoot deformity, but the limitations of these scoring systems are recognised (6). Foot creases are not often found in the walking child. Scoring systems for the walking child with previously treated clubfoot have been developed, but these are outcome assessment tools and do not direct treatment.

The aim of this study is to develop a scoring system to evaluate the severity of delayed presenting clubfoot and to assess whether this can be used to predict the response to Ponseti casting in the walking child from 2 to 10 years of age.

## Patients and methods

Institutional review board approval was given to undertake this observational study which has therefore been performed in accordance with the pertinent ethical guidelines (i.e. Declaration of Helsinki, as laid down in 1964 and revised in 2008). There were no conflicts of interest for any author. All clinical pictures and videos taken for the purpose of this manuscript were with the written permission of parents or guardians.

### Scale development cohort

Using all the variables from both the Pirani [7] and the Diméglio [8] scoring systems for infant clubfeet, we set out to initially investigate the presence and severity of each of the clinical features in delayed presenting clubfeet. We also assessed interobserver agreement between 4 observers (TRN, ME, TT and ROEG). Assuming a correlation coefficient under the null hypothesis and alternative hypothesis as 0.2 and 0.6 respectively, power of 0.8 and an alpha of 0.05, the estimated minimum sample size was 37. We scored 42 consecutive clubfeet in untreated congenital idiopathic cases (26 patients). Demographics are shown in Table 1. We also assessed intra-observer repeatability by performing evaluations of 38 feet on 2 occasions prior to any treatment by the same 4 observers. There was a minimum of 4 weeks between assessments.

**Table 1:** Demographics of the patient groups for both the scale development and the testing cohorts

	Scale development cohort	Testing cohort
Number of feet	42	100
Number of patients	26	62
Sex (M/F)	18/8	38/24
Number of children with bilateral clubfeet (%)	16 (62%)	38 (61%)
Mean age (range)	6 (2–10)	6.5 (2–10)

**Fig. 1:**
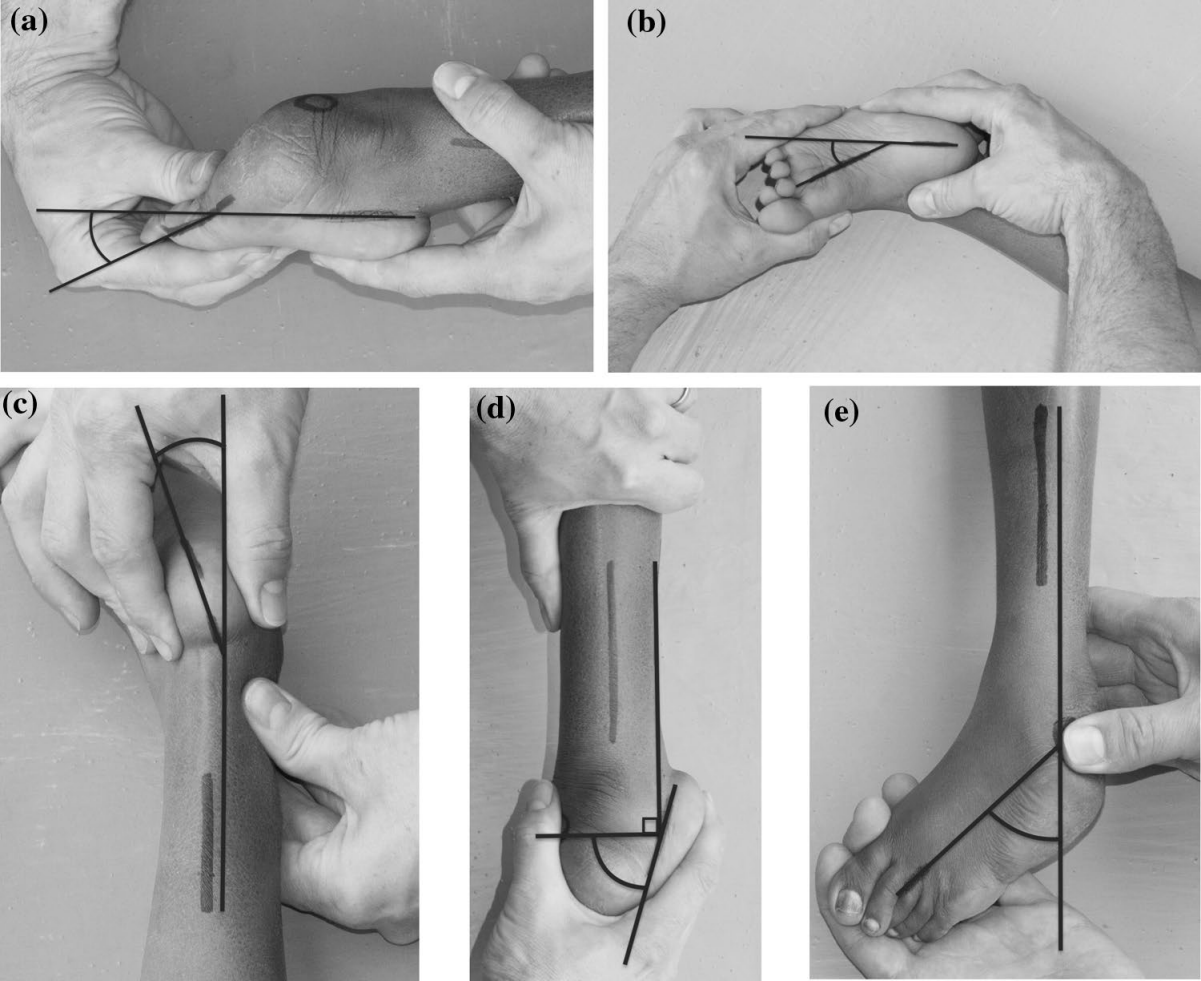
Worked example. Gentle corrective force is applied whilst measuring the angles with a goniometer. To begin with this is best done using 2 people. Plantaris 25 degrees = 2 points, adductus 27 degrees = 2 points, varus 18 degrees = 1 point, equinus 72 degrees = 3 points, rotation around talar head 44 degrees = 2 points, P + A+V + E+R = 10 points. Child is 8 years old—multiplier = × 2. PAVER score is 2 × 10 = 20/30

In distinction to the infant clubfoot, it was found that the elements of the ankle equinus and the foot plantaris could be differentiated from each other clinically (Fig. 1a, d) in delayed presenting clubfeet. These elements were separately recorded. All angles were recorded using gentle manipulation to reduce the deformity in the plane being assessed with a goniometer. Raters did not derive or combine scores from each scale to calculate the total scores at the time of recording. Raters could choose whether they used an assistant to help hold and manipulate the foot whilst it was being measured.

### Consensus group and agreement

The raters who are expert and experienced in delayed presenting clubfoot management discussed which factors should be included in the score. Criteria for inclusion were elements from these scoring systems that were present in the delayed presenting clubfoot and that had good reliability and discrimination. In order to widen the range of the scale, elements that were always present in the delayed presenting clubfoot were not discriminatory and were not included further. Intra-class coefficients, Cohen’s Kappa and Cohen’s Kappa with Landis–Koch extension and intra-class correlation were used to assess association and agreement as appropriate. Agreement was classified according to Cicchetti [9]. Cronbach’s alpha was assessed for the elements comprising the final scale. StatsDirect V3 was used for statistical analysis.

We wanted to see whether the score could be used to predict ease of correction. From experience, we had learned that an older child requires more casting and we agreed that it was important to incorporate a multiplier variable according to the age of the child into our final score. As all elements are made stiffer with age, it was helpful to multiply the combined elements by this factor.

### Testing cohort

The score was prospectively assessed in practice over the course of 11 months on 113 consecutive delayed presenting clubfeet in 69 children. Neurogenic and syndromic talipes were excluded (7 children), leaving 100 delayed presenting idiopathic clubfeet in 62 patients. Demographics are shown in Table 1. Responsiveness of the score was assessed longitudinally; we assessed the deformity score with the total number of casts required to achieve full correction as the primary clinical outcome. This included outpatient and any cast changes in hospital. The clinicians and physiotherapists performing the castings were unaware of the total scores.

Criterion validity was assessed by comparing the score with two validated outcome scoring systems for delayed presenting clubfeet. The patient or parent reported Roye score [10] and the Bangla score [11] that combines assessments of aesthetics, symptoms, functional abilities and clinical examination elements. The question on treatment satisfaction was not included as no patient had yet received treatment. For the reporting of functional scores, bilateral clubfeet were analysed alone as it is recognised that functional disability of bilateral feet is worse than unilateral [12].

Patients were assessed by pedobarography (EMED ST4, Novel^®^GmbH, Munich, Germany). The pedobarographic method was standardised according to Sinclair et al. [13]. The average of five representative recordings during normal unaided walking was used. All pedobarographic indices were expressed as a percentage of what a normal foot should be, for a given length. To achieve this, a series of 75 normal feet were assessed as controls. The control group was taken from children from the same population who attended with conditions that did not affect their lower limb or foot shape or function. The relationship between foot length and normal footprint areas and pressures was determined graphically. Using a trend line, the normal relationship between foot length and the peak pressure and footprint area could be identified. Foot pressures were expressed as a multiple of the normal and footprint area as a percentage of normal.

### Patient treatment protocol

All patients received casting according to the Ponseti principles by physiotherapists under the supervision of TRN. The aim of the treatment was correction of midfoot deformity with talar head coverage and hindfoot varus to at least neutral posture. Cavus was addressed first and casting continued to address abnormal cavus as long as it was present. Thereafter, rotation around the talar head was performed. In distinction to the Ponseti technique in infants, casts were changed every 2 weeks. Patients were treated in long leg casts with the knee at 45 degrees of flexion. This helped to reduce knee stiffness and was able to control rotation adequately. Significant moulding was applied using the thenar eminence rather than the thumb pulp for talar head pressure in larger feet to prevent areas of high pressure. An additional 5 min of pre-casting manipulation was performed. Up to a maximum of 9 outpatient casts were applied, corresponding to 4.5 months. This was restricted pragmatically to limit a prolonged casting phase.

**Fig. 2:**
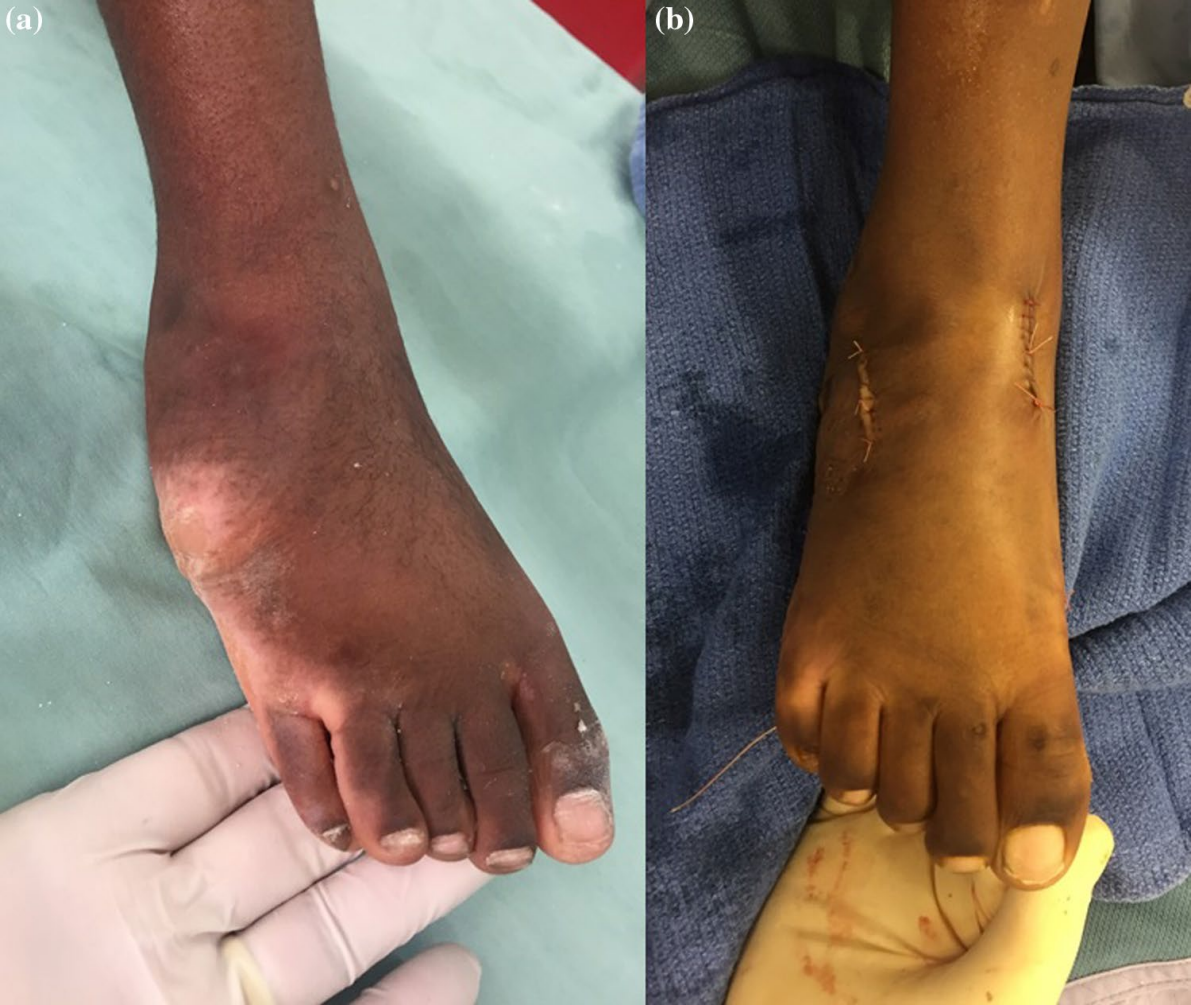
Clinical picture of a right foot in an 11-year-old. The midfoot correction was achieved following 9 casts. Equinus correction was achieved after a percutaneous Achilles tendon lengthening followed by a cast wedge. This illustration shows cuboid prominence (**a**) and the post-surgical appearance (**b**) following tibialis anterior tendon transfer to the lateral cuneiform with additional cuboid decancellation performed

Once the midfoot was corrected and the heel varus at least corrected to neutral alignment, the child was admitted to surgery. Talar head coverage was confirmed radiologically if this was uncertain clinically. Surgery consisted of a percutaneous 3-step Achilles tendon lengthening using a Hoke technique as described by Bleck [14]. Initially, 6 of the feet received a tibialis posterior musculo-tendinous lengthening with plantar fascia releases; however, this procedure was not used for the rest of the patients in this series as it was not found to be beneficial. The ankle and subtalar joints were not violated. Dorsiflexion to a minimum of 15 degrees was achieved using cast wedging as needed. We have found this to be a more predictable and effective procedure than posterior ankle capsulotomy and also avoids deep scarring. The total numbers of cast changes (including cast dorsiflexion wedging) were recorded. Once a minimum of 15 degrees was achieved, patients over the age of 3 years had a full tibialis anterior tendon transfer to the lateral cuneiform (95% of feet). This was secured through a drill hole and a button to the sole of the foot. A tendinous distal abductor hallucis release was also performed. These procedures were performed to reduce the risk of recurrence as no patient used a day-time ankle foot orthosis (AFO). A night AFO was used for 6 months for selected cases only. An additional cuboid decancellation (Fig. 2a, b) was included if the cuboid looked prominent clinically after the foot was fully corrected (8% of feet). This was performed on selected older children in this cohort at the same time and through the same incision as the tibialis anterior tendon transfer.

Failure of cast treatment was defined as children who had received 9 outpatient casts but still had an uncorrected midfoot in any plane. In these children, treatment resorted to more traditional, joint sacrificing surgical approaches, or soft tissue distraction using the Ilizarov apparatus.

In children who had treatment, the severity score and age were compared to the total number of cast changes. Comparison was also made of Bangla score, Roye score, pedobarographic indices and total number of cast changes. Kendall’s tau coefficient was used to calculate the rank correlation coefficients, and 2-sided *p* values were used. *p* < 0.05 was deemed statistically significant. Receiver operating characteristic (ROC) analysis determined the optimal cut-off score for casting failure.

## Results

### Scale development cohort

All of the variables of the Pirani and Diméglio scores were assessed for their inclusion. In our cohort, no patient had a posterior crease; only 1/42 had a significant plantar crease (aged 3 years). All delayed presenting feet had a pronounced curved lateral boarder. These variables were not discriminatory in the delayed presenting clubfoot. It was also difficult to quantify cavus clinically without standardised radiographs. However, forefoot plantaris, as measured from the lateral border, was easy to assess and the lateral calcaneal border was easy to define as it coincided with the junction of the glabrous sole skin and the dorsal foot skin (Fig. 1). The variables of calf wasting, presence of the empty heel and the talar head coverage had poor agreement between observers (Table 2).

**Table 2:** Variability and repeatability of aspects of clubfoot assessment tools expressed as Kappa values

	Inter-observer	Intra-observer
Hindfoot varus	0.67	0.70
Ankle equinus	0.53	0.78
Adductus	0.54	0.56
Rotation around talus	0.66	0.59
Plantaris	0.55	0.64
Empty heel	0.25	0.22
Talar head coverage	0.21	0.28
Gross calf muscle wasting	0.30	0.31

### Consensus group and agreement

**Fig. 3:**
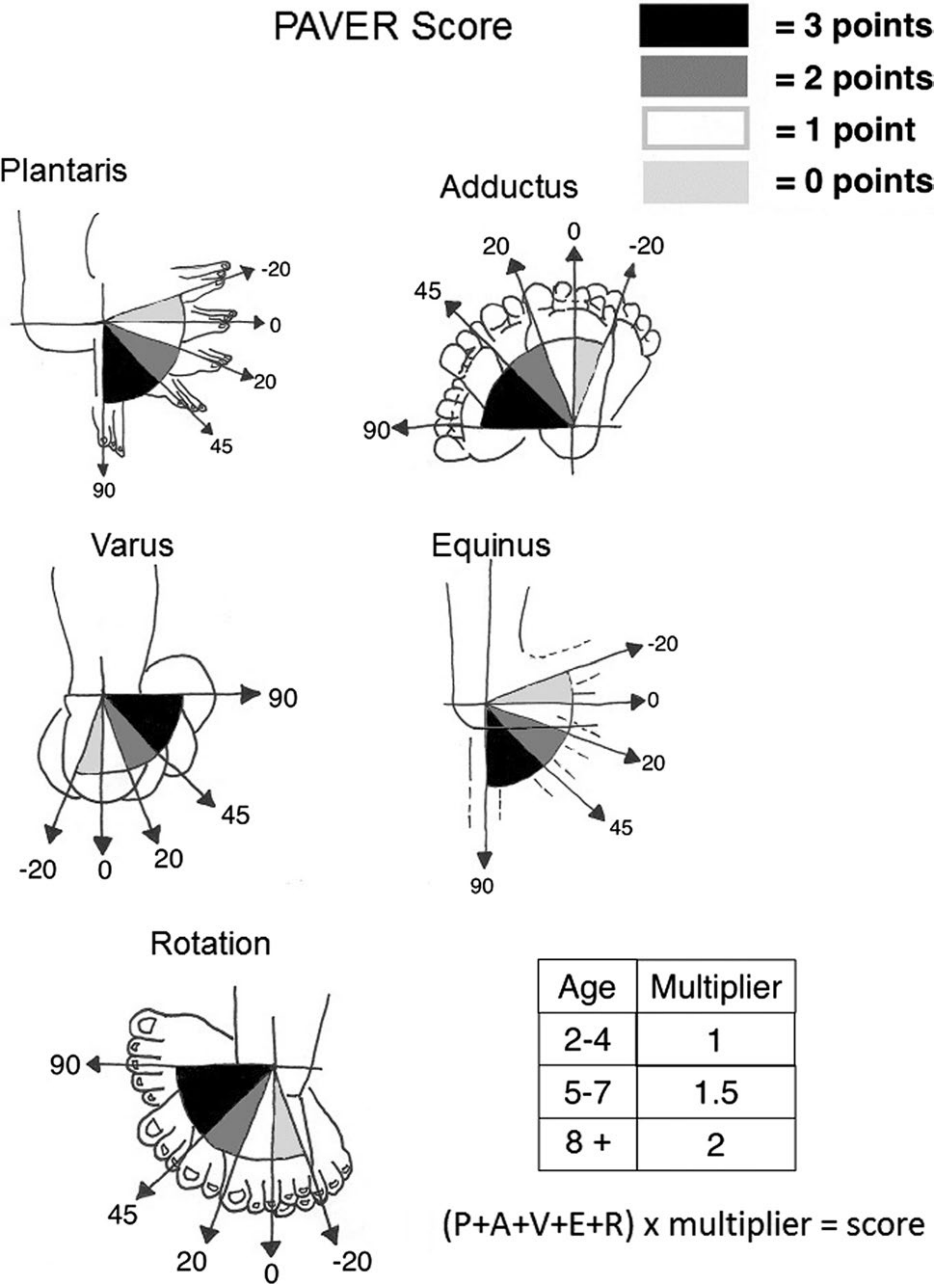
Schematic diagram of the score elements and the final calculation

**Fig. 4:**
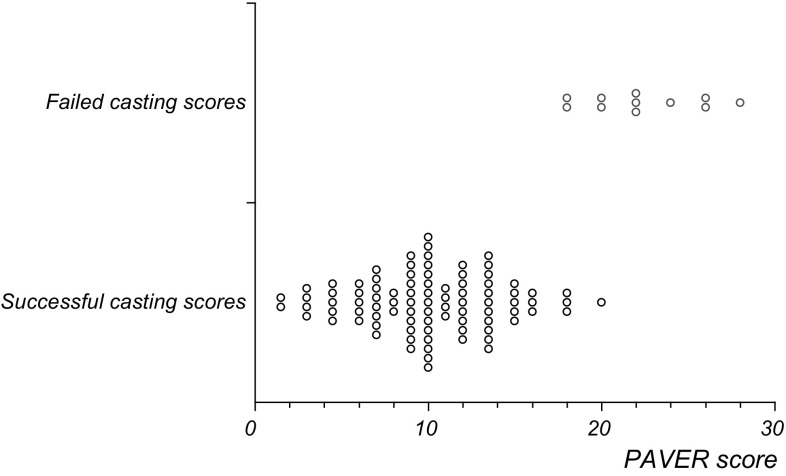
PAVER scores according to success or failure of casting

Five deformities were selected as being important for inclusion in our score with good discrimination and reliability. These comprised plantaris of the foot, adductus of the midfoot, varus of the hindfoot, equinus of the ankle joint and rotation around the prominent talar head (PAVER). A video is attached to view a demonstration of the measurement technique of the 5 angles (video 1). Additional informed consent was obtained from all individual participants for whom identifying information is included in this article. These measured angles were converted to points by using a simple algorithm (see Fig. 3). Correction to < 0 degrees scored 0 points, 0–20 scored 1 point, 21–45 scored 2 points and > 45 scored 3 points. The 5 deformity scores were added to give a total score between 0 and 15 points (Fig. 3). Cronbach’s alpha score was 0.76 demonstrating acceptable internal consistency. Total scores had an intra-class correlation coefficient among the 4 observers of 0.55. Total scores were grouped into mild (scores 1–5), moderate (6–10) and severe deformities (11–15) with a very good intra-observer variation of (Kappa = 0.89) and interobserver variation (Kappa = 0.92). We decided that age should form a significant part of the PAVER score. We agreed that the multiplier should increase according to age brackets 2–4, 5–7 and 8–10 years of age. Simple 1, 1.5 and 2 multipliers for ages 2–4, 5–7 and 8–10 years, respectively, were incorporated. The PAVER score is therefore calculated by adding up the component deformity points and multiplying by factor according to age which gives a maximum score of 30 (Fig. 4).

### Testing cohort

Using the developed score, 100 delayed presenting clubfeet were prospectively assessed. The distribution of the total score was 1–28, mean of 11.5/30. Casting with a limited surgical approach as described was successful in 89% of the cases. Out of 100 feet, there were 6 feet in 4 patients that we deferred cast treatment owing to social reasons, so an additional 6 feet were substituted. It was possible to measure every case, and the assessment can be performed in under 2 min. Prone positioning of the patient was helpful for all assessments apart from rotation around the talar head. The knee was positioned in flexion for assessment of equinus.

The total score in bilateral cases was correlated with higher Roye score and worsening Bangla score (tau = 0.32, − 0.37, respectively, both *p* < 0.05). The total score was correlated with higher peak pressures under the foot (tau = 0.36, *p* < 0.05) and was negatively correlated with total footprint area expressed as a percentage of expected (tau = − 0.39, *p* < 0.05). The severity score was positively correlated with the total number of casts to achieve a full correction (tau = 0.53, *p* < 0.05). There was a fair correlation between age and the number of casts needed for correction (tau = 0.43, *p* < 0.05). Poor correlation existed between age and score severity (tau = 0.12, *p* = 0.1), indicating age to be an independent factor. The PAVER score which includes the age multiplier had a good association with total cast number (tau = 0.71, *p* < 0.05). Adjusting the multiplier by reducing or increasing the effect did not increase the association of these variables.

Using ROC analysis, giving equal importance to sensitivity and specificity, set the maximum cut-off multiplier score for casting and limited surgery at 18/30. The probability of failure with a score of > 18 was 90%, and the probability of success if the score was 18 or less was 97%. Only one patient who had a PAVER score over 18/30 was fully corrected (Fig. 3).

## Discussion

A delayed presenting clubfoot score has been developed that can offer an excellent indication of outcome following manipulation and a modification of the Ponseti method. A score of 18 or below was associated with a high success rate of casting.

We noted that younger children responded better to manipulation and casting. We believe this is due to the favourable visco-elastic properties of soft tissues in the younger child. We did notice on fluoroscopy on completion of casting that the clubfoot bony architecture and the talo-navicular alignment was satisfactory in the corrected feet (although not normal). Up to the age of 11, score severity was not associated with age. Our experience with more mature feet over the age of 12 is different. Deformity becomes rigid in the idiopathic clubfoot at maturity, and casting alone cannot correct the stiff midfoot. Our observation in the older foot it is that all deformities tend to become rigid and that the plantaris in particular tends to become more pronounced. We believe that the score will remain appropriate for children 11 years and older particularly as plantaris is one of the components of this score. This will require further investigation.

The PAVER score is a severity score that uses gentle manipulation and passive correction which does account for flexibility of the foot. Clubfoot in a patient with increased ligamentous laxity and joint mobility would score lower and be expected to respond to casting faster than a more stiff foot. The PAVER score performed at initial assessment is likely to account for individual variation in ligamentous laxity.

Casting and a limited surgical approach is joint sparing. Along with other authors, we believe that this reduces complications compared to using an invasive approach and is likely to provide the best results retaining as much flexibility of the foot as possible [6, 16]. Long-term follow-up results are required to establish this. Outcome measures of these patients are continuing. We would therefore advocate the consideration of casting first for these patients. It must be emphasised that the casting protocol differs from standard Ponseti casting as outlined above but remains true to the Ponseti principles of sequentially addressing deformities starting with cavus and ending with correction of ankle equinus. Our patients also do not have day-time ankle foot orthotics but receive a tibialis anterior tendon transfer as a stabiliser. Reports from India [15] and from the Brazilian and Nepalese contexts [16, 17] are encouraging cast treatment for delayed presenting clubfoot up till the age of 10. Non-published reports from other African contexts (Mercy Ships), CURE Niger and immigrant populations to Scandinavia also report success using a similar casting technique (personal communication, Veltjens, Negrini and Klungsøyr).

Scores were assessed at the end of 9 cast changes. In cast responsive feet, equinus was the only deformity element yet to be corrected. There were 11 feet that were cast resistant. Five feet had remaining cavus (in addition to the other deformities) that would not respond significantly, and 6 had residual adductus, varus and rotation with cavus-corrected. Using the score for monitoring during casting would be an avenue for further investigation and helpful to define test responsiveness. Optimal treatment of delayed presenting resistant clubfeet that fail casting has not been defined in outcome-based studies. This is now underway as a study for those that failed casting. For those that have a PAVER score of over 18, we are investigating different surgical treatment options.

Our study has limitations to highlight. Although we were careful to ask about patient’s age, this was not known precisely for many patients as no birth certificate system is operated in Ethiopia at the time of this study. No advantage was gained in falsifying the age given by parents in this group, so we feel it remains reasonable to use the age parents reported.

Association with the Roye score and the Bangla outcome scores was mild. These scores were designed for use with post-treatment plantigrade feet. Our patients were pre-treatment and often very severe in bilateral cases. These mostly scored highly, and this exposed a significant ceiling effect of these two tools in using them for a pre-treatment purpose. This highlights the need for a score that is validated for pre-treatment cases.

We found that the very severe clubfeet often develop a dorsal skin pad from weight bearing. Peak pressures from this are sometimes less than some feet which have mild supination, resulting in lateral border weight bearing only, and the formation of point callosities, usually over the 5th metatarsal skin. This was exemplified in one bilateral asymmetric case where the pressures were opposite to the severity, i.e. the less severely affected side having higher pressures. This probably explained why the associations with pedobarographic indices were not stronger. The pedobarograph was also unable to automatically identify the clubfoot as it was unusual for there to be any toe contact. This tool may be more useful for post-treatment comparative assessments.

## Conclusion

The study shows that the PAVER score which is comprised of deformity assessment with an age-specific multiplier is a valid tool for use in the delayed presenting clubfoot. Clinically, its predictive value can be used to help determine whether casting and a limited operative approach could be successful in a child 10 years or younger. It could be further used as a research tool for describing degree of delayed presenting clubfoot deformity and for comparing treatments of like deformities.

## Electronic supplementary material

Below is the link to the electronic supplementary material.

Supplementary material 1 (MP4 450948 kb)
